# Imaging Characteristics of USPIO Nanoparticles (<5 nm) as MR Contrast Agent *In Vitro* and in the Liver of Rats

**DOI:** 10.1155/2019/3687537

**Published:** 2019-07-21

**Authors:** Xiaohong Ma, Shuang Wang, Longbin Hu, Shichao Feng, Zhiyuan Wu, Siyun Liu, Shaofeng Duan, Zhongwei Chen, Chunwu Zhou, Xinming Zhao

**Affiliations:** ^1^Diagnostic Radiology, Cancer Hospital, Chinese Academy of Medical Sciences & Peking Union Medical College, Beijing 100021, China; ^2^State Key Laboratory of Molecular Oncology, Cancer Hospital, Chinese Academy of Medical Sciences & Peking Union Medical College, Beijing 100021, China; ^3^GE Healthcare (China), Beijing 100176, China

## Abstract

Iron nanoparticles have an increasingly more and more important role in MR molecular imaging due to their novel magnetic and surface chemical properties. They provide new possibilities for noninvasive diagnosis and treatment monitoring, especially for tissues that are rich in macrophages. The smaller size and prolongation of the plasma half-life change the *in vivo* fate of ultrasmall superparamagnetic iron oxide (USPIO) nanoparticles captured by liver in reticuloendothelial system (RES) or mononuclear phagocytic system (MPS). However, there is still a lack of MR imaging studies on the liver assessing USPIO nanoparticles <5 nm in size to reflect its absorption and clearance properties. In this study, we used MRI to study the *in vitro* phantom and *in vivo* rat liver imaging characteristics of USPIO nanoparticles (<5 nm). The results showed that USPIO nanoparticles (<5 nm) could potentially reduce longitudinal and transverse relaxation times and showed similar *T*_1_ relaxation rates compared with commercial gadolinium chelates. In addition, USPIO nanoparticles (<5 nm) *in vivo* demonstrated both positive (*T*_1_) and negative (*T*_2_) liver contrast enhancement in healthy rats' liver. Furthermore, USPIO nanoparticles showed relatively good in vitro biocompatibility and fast clearance (within 45.17 minutes after intravenous injection) in the normal liver. Taken together, these data might inspire a new personalized and precise diagnostic tool and stimulate new applications for specific targeted molecular probes.

## 1. Introduction

Magnetic resonance imaging (MRI) is widely used in clinical practice due to its advantages of nonionizing radiation, multisequencing, and better soft tissue contrast. The latest results of molecular imaging using MR scanning provide new methods for noninvasive detection or tracking of such conditions as hepatocellular carcinoma [1, 2], atherosclerotic plaque [3], collateral circulation in acute ischemic stroke [4], and glioma gene therapy [5]. It is also emerging as a kind of functional MR probe system, for example, stimuli-responsive MRI-monitored drug delivery system, including pH-responsive [6–8] or thermo-responsive [9] ones. Among diverse MR molecular imaging studies, superparamagnetic iron oxide (SPIO) nanoparticles have played an important role [10, 11].

SPIO nanoparticles have been approved by the U S Food and Drug Administration (FDA) and European Commission for use as a type of MRI contrast agent [12]. They are attracting intensive attention and have many potential applications in MRI because of their novel magnetic properties and surface chemistry for ligand binding and biosafety optimization [13–15]. In contrast from gadolinium-based contrast agents, SPIO nanoparticles showed stronger magnetic susceptibility and size- or surface-dependent pharmacokinetics and image features [16, 17]. In addition, the low cost, biological safety, and flexible surface modifications promote wide utilization of SPIO nanoparticles as contrast agents or as a platform for construction of specific targeting probes in MRI research.

SPIO nanoparticles are typically classified by their hydrodynamic diameter into three categories, which are oral (large) SPIO nanoparticles at 300 nm to 3.5 *μ*m, standard (regular) SPIO (SSPIO) nanoparticles at 50 to 150 nm, and ultrasmall SPIO (USPIO) nanoparticles less than 50 nm [18, 19]. Some differences between SSPIO and USPIO nanoparticles have been reported. First, larger SPIO nanoparticles show higher nonspecific uptake by the mononuclear phagocyte system (MPS) or reticuloendothelial system (RES) compared with smaller USPIO nanoparticles, which indicates a higher percentage of passive uptake of larger particles for tissues rich in macrophages, such as the liver, spleen, lymph nodes, or bone marrow [19]. Secondly, unlike most SSPIO nanoparticles, which predominantly enhance the transverse relaxation rate (1/*T*_2_) and function as negative contrast agents, some USPIO nanoparticles with smaller core size can enhance both longitudinal (1/*T*_1_) and transverse relaxation rates [20]. Furthermore, USPIO enhancement effect on *T*_1_ relaxation rate is stronger with decreased particle size and core size (∼5 nm) has been suggested to be optimal positive (*T*_1_) contrast agent benefit from the enhancement of *T*_1_ and suppression of *T*_2_ [17, 21–24]. Thirdly, USPIO nanoparticles with smaller diameters have shown better biosafety. Faster biodegradation rates in the liver and spleen have been recently reported for monodisperse 5 nm iron oxide cores in comparison with 15 and 30 nm iron oxide cores coated with the same coating molecules [25].

Therefore, we hypothesize that USPIO nanoparticles, especially ≤10 nm, have significant potential as positive contrast agents [26–28]. Smaller USPIO nanoparticles may also herald a novel iron-oxide-nanoparticle-based imaging technique for tissues that are rich in macrophages, such as the liver. Significant increasing publications have adopted USPIO nanoparticles that are approximately 10 nm in size as the platform for construction of targeted probes that are specific to the liver tumor [1, 29–32]. However, most of these studies still made use of *T*_2_ effect of USPIO nanoparticles. Meanwhile, there is still a lack of investigation of *in vivo* liver uptake and liver MR imaging properties of USPIO nanoparticles that are <10 nm in size [2, 33–37], although the potential of USPIO nanoparticles with smaller core size as positive contrast agent for liver angiography and focal lesion detection has emerged gradually during clinical studies [2, 36].

Therefore, in this preliminary study, we mainly investigated the phantom imaging properties of USPIO nanoparticles (<5 nm) *in vitro* and the imaging feature in the liver of rats using MRI. The phantom was designed to study the small USPIO nanoparticle characteristics of image enhancement. Healthy Wistar rats were used to study the biodistribution of USPIO nanoparticles (<5 nm) in the liver using a 3.0 Tesla clinical MR scanner. The study could be used as a reference for USPIO-based specific or targeted probe design for liver focal lesions or tumors.

## 2. Materials and Methods

USPIO solution (5 mg/ml) was purchased from Sigma-Aldrich (Catalog 725331, St. Louis, MO, USA). Wistar rats (average weight: 180 g, sex: male) were purchased from Charles River Laboratories (Beijing Vital River Laboratory Animal Technology Co., Ltd). The rats were kept in clean grade animal room of Cancer Hospital at 23 ± 1°C, with free access to water/food and 12 hours light/dark cycle. All experiment protocols were approved by the Animal Care and Use committee of Cancer Hospital, Chinese Academy of Medical Sciences (CAMS). All experiments were conducted in accordance with the Animal Guidelines of CAMS.

### 2.1. USPIO Nanoparticle Morphology

The morphology, average size, and size distribution of USPIO nanoparticles were characterized by transmission electron microscopy (TEM; FEI Tecnai G2 F30F30, USA) with acceleration voltage of 300 kV. TEM samples were prepared by dropping the USPIO solution onto 400 mesh copper grids with carbon film.

### 2.2. Phantom Imaging

To investigate the MR imaging characteristics of USPIO nanoparticles, the phantom was constructed by USPIO saline solutions (0.9% sodium chloride) with gradient concentrations, which were contained in individual wells (300 *μ*L) of 96-well plate. The iron concentration of USPIO solution was 1.5, 1, 0.5 0.25, and 0.1 mM, respectively.

Phantom MR imaging was performed on 3.0 Tesla clinical MR scanner (750 W, GE Healthcare, USA) with 8-channel head coil. The *T*_1_ relaxation times were measured by IR sequences with a fixed echo time (TE) of 7 ms and repetition time (TR) of 2000 ms and multiple inversion time (TI) of 700, 500, 400, 300, 200, 150, 100, 80, and 50 ms. *T*_2_ images were acquired using spin echo (SE) sequence with different TE ranging from 10 ms to 170 ms. The parameters were set as follows: TR = 2000 ms, TE = 10, 20, 30, 40, 50, 70, 90, 110, 130, 150, and 170 ms, matrix = 256 × 256, field of view (FOV) = 20 mm × 20 mm, and slice thickness/slice separation = 3 mm/3.3 mm, and NEX = 2.0.

### 2.3. Cell Culture

The rat normal hepatic cell line BRL-3A (CASC040; Shanghai Jian Blunt Biological Technology Co., LTD, Shanghai, China) was chosen for *in vitro* experiments. BRL-3A cells were cultured in RPMI-1640 supplemented with 10% fetal bovine serum (FBS) and 1% penicillin-streptomycin at 37°C in a 5% CO_2_ atmosphere.

### 2.4. MTT Assay

BRL-3A cells were seeded in a 96-well cell plate at a density of 3 × 10^3^ cells/well and allowed to attach to the bottom well for over 12 hours. Every five repeated wells containing adherent cells were exposed to USPIO nanoparticles at different concentrations (0, 20, 50, 100, 200, and 400 *μ*g·Fe/ml) and then incubated at 37°C in a 5% CO_2_ atmosphere for 12 and 24 hours. After the incubation, the cells were washed with phosphate buffered saline (pH = 7.4) and completely removed, followed by theaddition of cell culture medium. Then, 20 *μ*L 5 mg/mL MTT solution (3-(4,5-dimethyl-2-thiazolyl)-2,5-diphenyl-2-H-tetrazolium bromide) was added per plate and the plates were incubated for another 4 h at 37°C in a 5% CO_2_ atmosphere. After 4 h incubation, the solution was removed and 150 *μ*L of dimethyl sulfoxide (DMSO) was added to each wall with shaking of the plate until the crystals were dissolved and turned from yellow to violet. The optical density (OD) value of each well at 490 nm was measured using a microplate reader (DNM-9602, Perlong Medical, Beijing, China).

### 2.5. Prussian Blue Staining Assay

Prussian blue staining was utilized to visibly assess the iron uptake by BRL-3A cells that were treated with USPIO nanoparticles with different concentrations (0, 20, 50, and 100 *μ*g·Fe/ml) for 6 and 12 hours. BRL-3A cells were seeded in 6-well cell plates at a density of 5 × 10^5^ cells in each well, and every two wells of cells were incubated with the same USPIO concentration of 0, 20, 50, and 100 *μ*g·Fe/ml separately for 6 h and 12 h. The cells were gently rinsed three times with 1x PBS and fixed with 4% paraformaldehyde for 20 min at room temperature. After washing 3 more times with ultrapure water, the resulting cells were incubated with Perls stain solution A of Prussian Blue Staining Kit (DJ00001, Leagene, China) for 15 min, washed with ultrapure water, and followed by counterstaining with with Nuclear Fast Red solution B of staining kit. The results of Prussian blue staining were assessed using bright-field optical microscopy.

### 2.6. In Vivo Characterization

Wistar rats were scanned using a 3.0 T MR device (GE Discovery 750) with a 4-channel animal coil. The rat was firstly anesthetized by tribromoethanol (Avertin) at a dosage of 500 mg/kg body weight via intraperitoneal injection, and the anesthesia could last for ∼50 min. The abdominal region of the rat was placed at the center of the coil, and the liver was the target. USPIO solutions were transferred into medical-grade physiological saline (0.9% sodium chloride, pH = 7.5) with final volume ∼200 *μ*L and ready for use. Prior to USPIO injection, *T*_1_WI and *T*_2_WI images were acquired, and this time point was defined as preinjection time, 0 s. Next, without moving the rat from animal coil, the USPIO solution was administered by tail vein injection at a dose of 1.8 mg·Fe/kg over 5 s and MR imaging was performed at 10 min, 15 min, 20 min, 25 min, 30 min 40 min, and 50 min after USPIO injection using the same MR sequence as for preinjection. The scanning parameters were described as follows. Axial *T*_1_WI/FSE with TR/TE = 475/10 ms, FOV = 60 × 60 mm, matrix = 512 × 512, resolution = 0.1172 × 0.1172 mm, slice thickness/slice separation = 0.8 mm/1.6 mm, and NEX = 4.0. Axial *T*_2_WI/FSE sequence with TR/TE = 1500/92 ms, FOV = 60 × 60 mm, matrix = 512 × 512, resolution = 0.1172 × 0.1172 mm, and slice thickness/slice separation = 0.8 mm/1.6 mm. The effect time and clearance time of USPIO nanoparticles in the liver were determined by signal-intensity (SI) *vs*. time curve, which was deduced from the series of *T*_1_-weighted images.

### 2.7. Statistics

The statistical analysis process was implemented on the R language (version 3.5.1, 2018) software, and the normality test was performed using the Shapiro–Wilk test. For the difference test of the viability characteristics of the same group of cells, when the test group and the control group all met the normal distribution, the paired *t*-test was used after the homogeneity test of the variance. When one of the test groups or the control groups did not satisfy the normal distribution, we used the Mann–Whitney *U* test in the nonparametric test. The *p* value in the test was compared to the significance level (*α* = 0.05).

## 3. Results

### 3.1. USPIO Core and Phantom Imaging

**Figure 1:**
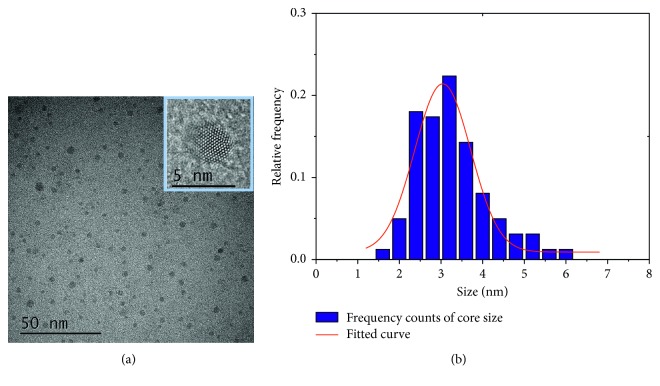
The core size distribution of USPIO. (a) TEM images of USPIO nanoparticles were utilized during the experiment. Inset: high-resolution TEM image of a nanoparticle; scale bar, 5 nm. (b) The core size distribution of USPIO nanoparticles with mean diameter, 3.26 nm, and standard deviation, 0.87 nm, determined from the TEM images.

**Figure 2:**
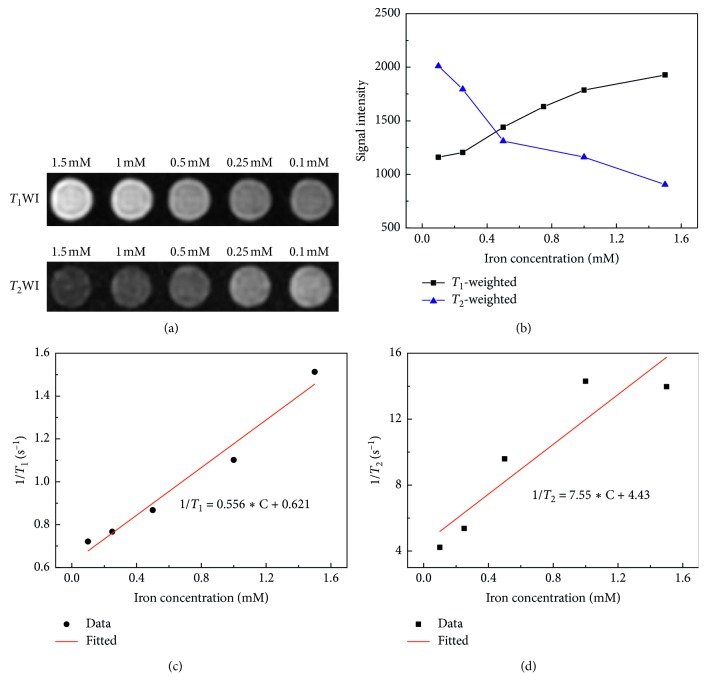
Relaxation profiles of USPIO nanoparticles with varying concentration. (a) *T*_1_-weighted and *T*_2_-weighted images of USPIO nanoparticles in saline solution (0.9% sodium chloride) with different iron concentrations from 0.1 mM to 1.5 mM. (b) The change of mean *T*_1_- and *T*_2_-weighted signal intensity as the iron concentration. (c) The relationship of longitudinal relaxation rate and iron concentration and the derived *r*_1_ relaxivity 0.556 mM^−1^·s^−1^. (d) The relationship of transversal relaxation rate and iron concentration and the derived *r*_2_ relaxivity 7.55 mM^−1^·s^−1^.

The core diameter of USPIO nanoparticles was <4.20 nm (3.26 ± 0.87 nm) as shown in TEM image (Figure 1(a)) and core size distribution analysis (Figure 1(b)), indicating significant potential as a positive contrast agent [29, 38–40]. It was found simultaneously from *T*_1_-weighted and *T*_2_-weighted images and signals of the phantom in Figures 2(a) and 2(b) a positive enhancement of the *T*_1_-weighted MR signal and a negative enhancement of the *T*_2_-weighted MR signal as iron concentration increasing.

The longitudinal relaxation time *T*_1_ and transversal relaxation time *T*_2_ were also calculated, and their relationship with iron concentration was plotted in Figures 2(c) and 2(d). *T*_1_ and *T*_2_ values were negatively correlated with iron concentration, suggesting that USPIO nanoparticles accelerated the recovery of net magnetization. By linear fitting the proton relaxation rates (*R*_1_ = 1/*T*_1_ and *R*_2_ = 1/*T*_2_) with respective to the iron ion concentrations according to equation (1) [41, 42], the molar relaxivities (*r*_1_: longitudinal relaxivity and *r*_2_: transverse relaxivity) at 3.0 Tesla were extracted as *r*_1_ = 0.556 mM^−1^·s^−1^ and *r*_2_ = 7.55 mM^−1^·s^−1^, respectively:

(1) $$\begin{array}{c} {\left(\frac{1}{T_{i}}\right)}_{\text{w}}={\left(\frac{1}{T_{i}}\right)}_{\text{wo}}+r_{i}\left[{\text{Fe}}_{3}{\text{O}}_{4}\right],\;\;\;i=1,2. \end{array}$$

### 3.2. In Vitro Biocompatibility of 5 nm USPIO Nanoparticles by MTT and Prussian Blue Staining Assay

**Figure 3:**
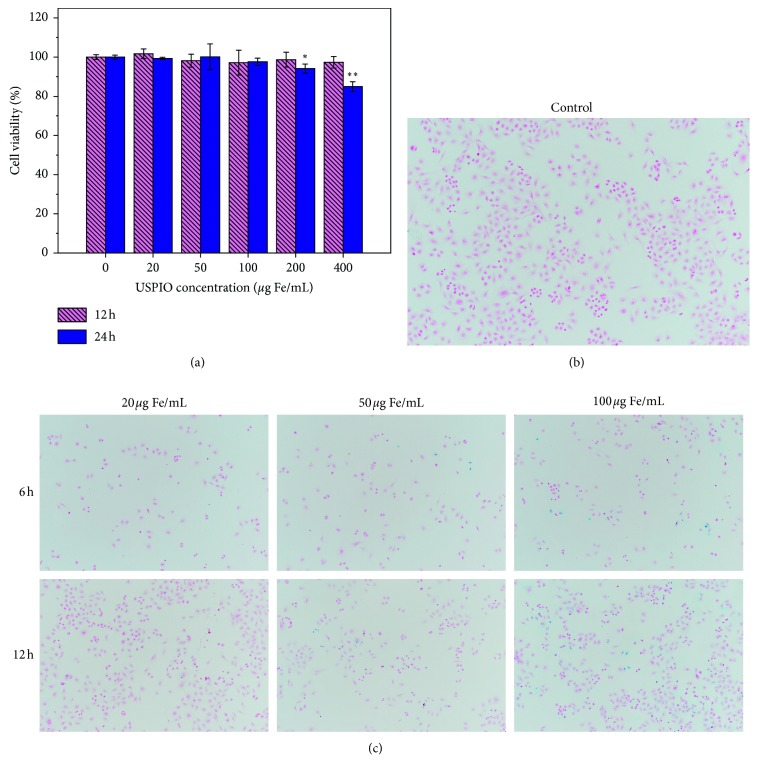
The influence of 5 nm USPIO nanoparticles on the cell viability and uptake of BRL-3A cells determined by MTT and Prussian blue staining assay. (a) Statistical analysis of MTT results revealed that only 24 hours incubation of 200 *μ*g·Fe/mL and 400 *μ*g·Fe/mL USPIO nanoparticles significantly weakened the cell viability as compared with the control (^*∗*^*p* < 0.05; ^*∗∗*^*p* < 0.001), respectively. For each group in MTT assay, 5 wells per plate were treated identically. (b, c) Cellular uptake of 5 nm USPIO nanoparticles determined by iron Prussian blue staining assay. Rat normal hepatic cells BRL-3A were treated by USPIO nanoparticles at 20, 50, and 100 *μ*g·Fe/mL for 6 and 12 hours, as compared with the control group treated by culture media.

The effect of 5 nm USPIO nanoparticles on the cell viability of rat normal hepatic cells BRL-3A was determined and quantified by MTT assay as shown in Figure 3(a). The 5 nm USPIO nanoparticles significantly reduced the cell viability to 94.17% and 84.9% at concentration of 200 *μ*g·Fe/mL for 24 hours incubation, while the cell viability maintained more than 95% for 24 h incubation at lower iron concentrations less than 200 *μ*g·Fe/mL or shorter incubation time (12 hours) at highest concentration of 400 *μ*g·Fe/mL. In addition, the Prussian blue staining (Figures 3(b) and 3(c)) also showed that the nonspecific uptake of USPIO nanoparticles by rat normal hepatic cells increased either as iron concentration or incubation time increased. However, the highest concentration of 100 *μ*g·Fe/mL in the iron staining experiment did not show very significant nonspecific uptake and influence on cell growth after 12 hours incubation.

### 3.3. In Vivo Characterization

**Figure 4:**
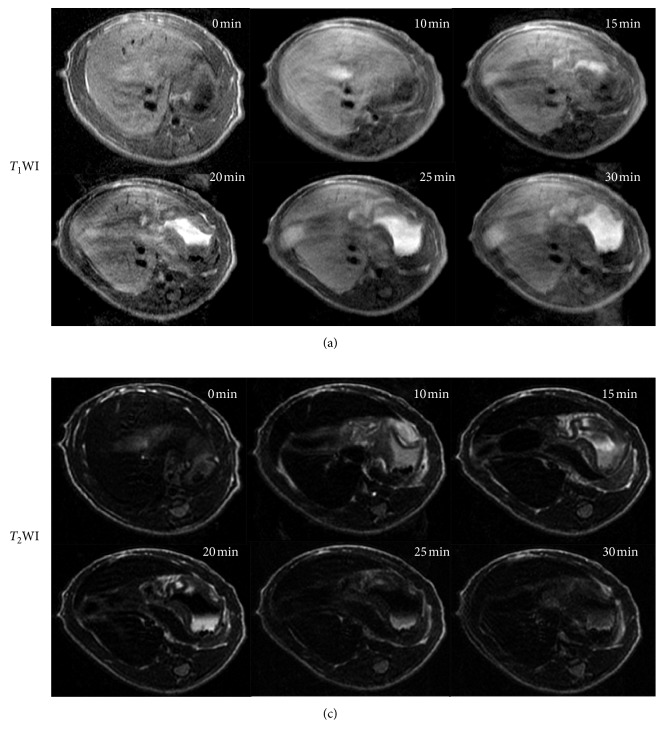
*T*_1_-weighted (a) and *T*_2_-weighted (b) images of the rat liver at 6 different time points (0 min, 10 min, 15 min, 20 min, 25 min, and 30 min).

*T*_1_WI and *T*_2_WI of the rat liver were generated at sequential time points before and after USPIO injection (pre-0 min, post-10 min, 15 min, 20 min, 25 min, 30 min, 40 min, and 50 min) to evaluate the uptake and imaging properties of USPIO nanoparticles (<5 nm; Figure 4). The *T*_1_/*T*_2_ contrast in the liver showed positive (negative) enhancement and started to recover within 30 min after injection. The half-life of USPIO nanoparticles in the blood and liver was 14.46 ± 2.21 min and 45.17 ± 4.50 min, respectively.

Based on these data, region of interest (ROI) data analysis was carried out to obtain regionally averaged *T*_1_(*T*_2_)-weighted signal intensity (SI) in the liver and paraspinal muscle, and the liver-to-muscle (L/M) ratios at each time point after USPIO injection were compared with preinjection ratio, as summarized in Table 1. We found that the positive *T*_1_ and negative *T*_2_ contrast appeared in liver immediately following injection, reaching their maximum at ∼10. The positive *T*_1_ contrast diminished at 45.17 min and was restored to approximately the same level prior to injection.

## 4. Discussion

Several commercial iron oxide nanoparticles have been approved by FDA for clinical applications [12]; however, their usage remains narrow, and they have not been well accepted in the field of radiology. This may be due to the traditional expression of negative contrast enhancement and their prolonged *in vivo* retention of up to several months. However, smaller USPIO nanoparticles have been implicated in the new routes for applications especially due to their beneficial safety profile, such as in nephrogenic sclerosis and for renal insufficient patients compared with gadolinium chelates. In addition, their slow phagocytosis from macrophages make them ideal candidates for tumor imaging in tissues that are rich in macrophages [12]. Therefore, we sought to explore the effect of using smaller USPIO nanoparticles, especially for liver imaging.

From *in vitro* phantom imaging findings, USPIO nanoparticles (<5 nm) possessed a similar *r*_1_ and higher *r*_2_ compared with conventional commercial gadolinium-based contrast agents (*r*_1_∼4 mM^−1^·s^−1^) [13, 14, 16], which means an effective and sensitive MR contrast enhancement capability [17]. These results indicated that in contrast with traditional iron oxide nanoparticles that are negative *T*_2_ contrast agents, USPIO nanoparticles (<5 nm) can function as simultaneous positive and negative MRI contrast agents. This was in accordance with the findings of other groups [2, 24, 36, 38], which could be attributed to the relatively larger number of Fe^3+^ with 5 unpaired electrons over the surface of smaller USPIO nanoparticles with larger surface area [40]. In addition, as the concentration of USPIO nanoparticles increased, there was an enhancement of the longitudinal and transversal relaxation rate, which suggested that they could be used in dynamic enhancement MRI, such as perfusion and permeability.

The *in vitro* biocompatibility experiments indicated a proper dosage during *in vivo* experiments. In our *in vivo* experiments, 5 nm USPIO nanoparticles were intravenously injected into rat at ∼1.8 mg·Fe/kg dosage, which could be translated into ∼26.4 *μ*g·Fe/mL blood volume [43]. Such dosage was well located in a safe range for a good biocompatibility and less nonspecific uptake by normal hepatic cells.

In addition, smaller USPIO nanoparticles at such *in vivo* dosage showed a good performance as positive (*T*_1_) and negative (*T*_2_) liver contrast agents simultaneously so that the absorption and clearance properties of USPIO nanoparticles in the rat liver could be derived from MR imaging data. For normal liver uptake, USPIO nanoparticles reached peak uptake at ∼10 min postinjection and were restored to preinjection levels at ∼45 min. The demonstration of such relatively fast clearance from liver compared with other commercial USPIO nanoparticles with ∼5 nm core size [35, 44] will have potential to facilitate an increased signal differential between tumor and normal tissues for tumor-targeted imaging when USPIO nanoparticles are labeled with specific tumor bio-markers. However, questions are still waiting for us to further explore during the basic research stage. For example, though such smaller USPIO nanoparticles show their potential as positive and negative contrast agents, how will their performance be as targeted probes when concentrated in tumors? Will the inhomogeneity of magnetic field brought by USPIO aggregation influence the performance of its longitudinal relaxation enhancement? In addition, are such smaller USPIO candidates in conjugation with multiple large-molecular-weight antibodies as targeted probes? Further research is needed for such kind of smaller USPIO nanoparticles.

## 5. Conclusion

In this paper, we investigated USPIO nanoparticles with a core size <5 nm. We assessed the phantom imaging features, biocompatibility properties *in vitro*, and time-dependent uptake and MRI properties *in vivo*. The results showed that smaller USPIO nanoparticles could potentially reduce longitudinal and transverse relaxation times. In addition, USPIO nanoparticles (<5 nm) showed good biocompatibility *in vitro* and both positive (*T*_1_) and very good negative (*T*_2_) liver contrast enhancement in healthy rats. Furthermore, USPIO nanoparticles showed relatively fast clearance (within 45.17 minutes after intravenous injection). Taken together, these may stimulate new applications for specific targeted molecular probes. Therefore, accompanied with development of surface chemistry technology and biomarker investigations, we argue that new developments in USPIO nanoparticles might inspire new personalized precise diagnosis.

## Figures and Tables

**Table 1:** The liver-to-muscle signal intensity ratio of the *T*_1_/*T*_2_-weighted image at different time points.

	0 min	10 min	15 min	20 min	25 min	30 min
*T*_1_ L/M^*∗*^	1.10	2.15	1.78	1.48	1.38	1.15
*T*_2_ L/M^*∗*^	0.45	0.30	0.34	0.35	0.43	0.43

^∗^Mean liver-to-muscle (L/M) ratio from two rats.

## Data Availability

The MR images and postprocessed data used to support the findings of this study are included within the article. Requests for the raw data, after publication of this article, will be available from the corresponding author upon request.

## Abbreviations

USPIO: Ultrasmall superparamagnetic iron oxide
SPIO: Superparamagnetic iron oxide
MR: Magnetic resonance
ROI: Region of interest
MPS: Mononuclear phagocyte system
RES: Reticuloendothelial system
*T*_1_WI: *T*_1_-weighted imaging
*T*_2_WI: *T*_2_-weighted imaging.
